# Role of Microbial Interactions across Food-Related Bacteria on Biofilm Population and Biofilm Decontamination by a TiO_2_-Nanoparticle-Based Surfactant

**DOI:** 10.3390/pathogens12040573

**Published:** 2023-04-07

**Authors:** Agapi I. Doulgeraki, Christina S. Kamarinou, George-John E. Nychas, Anthoula A. Argyri, Chrysoula C. Tassou, Georgios Moulas, Nikos Chorianopoulos

**Affiliations:** 1 Institute of Technology of Agricultural Products, Hellenic Agricultural Organization-DIMITRA, S. Venizelou 1, 14123 Lycovrissi, Greece; 2 Department of Molecular Biology and Genetics, Democritus University of Thrace, 68100 Alexandroupolis, Greece; 3 Laboratory of Microbiology and Biotechnology of Foods, Department of Food Science and Human Nutrition, School of Food and Nutritional Sciences, Agricultural University of Athens, 11855 Athens, Greece; 4 Moulas Scientific, Messinias 14, 15234 Chalandri, Greece

**Keywords:** microbial interactions, foodborne pathogens, biofilm, disinfection, TiO_2_ nanoparticles

## Abstract

Microbial interactions play an important role in initial cell adhesion and the endurance of biofilm toward disinfectant stresses. The present study aimed to evaluate the effect of microbial interactions on biofilm formation and the disinfecting activity of an innovative photocatalytic surfactant based on TiO_2_ nanoparticles. *Listeria monocytogenes*, *Salmonella* Enteritidis, *Escherichia coli*, *Leuconostoc* spp., *Latilactobacillus sakei*, *Serratia liquefaciens*, *Serratia proteomaculans*, *Citrobacter freundii*, *Hafnia alvei*, *Proteus vulgaris*, *Pseudomonas fragi*, and *Brochothrix thermosphacta* left to form mono- or dual-species biofilms on stainless steel (SS) coupons. The effectiveness of the photocatalytic disinfectant after 2 h of exposure under UV light on biofilm decontamination was evaluated. The effect of one parameter i.e., exposure to UV or disinfectant, was also determined. According to the obtained results, the microbial load of a mature biofilm depended on the different species or dual species that had adhered to the surface, while the presence of other species could affect the biofilm population of a specific microbe (*p* < 0.05). The disinfectant strengthened the antimicrobial activity of UV, as, in most cases, the remaining biofilm population was below the detection limit of the method. Moreover, the presence of more than one species affected the resistance of the biofilm cells to UV and the disinfectant (*p* < 0.05). In conclusion, this study confirms that microbial interactions affected biofilm formation and decontamination, and it demonstrates the effectiveness of the surfactant with the photocatalytic TiO_2_ agent, suggesting that it could be an alternative agent with which to disinfect contaminated surfaces.

## 1. Introduction

The theory that microbes can adhere to a surface was proposed in the early 1930s [1,2]. The history of this phenomenon had been well reviewed earlier [3]. The formation of a biofilm, i.e., cells enclosed in an extracellular polymeric matrix, alters cellular metabolism and, subsequently, a cell’s phenotype [4,5,6,7,8,9]. Biofilms have been detected on various industrial and non-industrial surfaces, and their composition and structure are affected by the different microorganisms that comprise their community [4,10,11,12,13]. Data on microscopic observations presented in the late 1960s revealed that the stability of one microbe strengthens the stability of the other species within a biofilm [14]. Considering the ability of microbes to communicate and co-exist in an environment, researchers noted that microbial interactions led to the development of biofilm communities [6,15,16,17,18,19,20,21].

The formation of a biofilm is of high importance in food-related environments, as the transmission of microorganisms in food may cause foodborne illness or food losses [9,22,23,24]. Indeed, the removal of *Escherichia coli*, *Listeria monocytogenes*, *Salmonella* spp., and *Campylobacter jejuni* from the industry is crucial [25,26,27]. The concern in this regard is the effective removal of the persistent, i.e., the remaining, microbiota after cleaning/disinfection [8,28,29,30,31]. However, the presence of more than one remaining species must be considered, as single-culture biofilms have been found to be more sensitive to disinfectants including peracetic acid and benzalkonium chloride [32]. Nowadays, new strategies have been developed to prevent the colonization and inhibit the growth of microorganisms, including nanotechnology and the use of essential oils, anti-quorum compounds, enzymes, etc. [16,33]. From the perspective of nanotechnology, nanoparticles can exhibit antibiofilm activity because of their penetration through a matrix [34,35,36]. The food industry seems to prefer the use of titanium dioxide nanoparticles (TiO_2_) as disinfectants compared to other metal oxides [37,38]. So far, the results have highlighted the ability of these nanoparticles to inactivate various microorganisms [35,39,40,41,42]. The combined action of TiO_2_ with ultraviolet light (UV) has been shown previously [40,43,44]. Kim et al. [40] observed that UV irradiation increases the effects of nanoparticles on the survival of *Listeria monocytogenes*, *Salmonella choleraesuis*, and *Vibrio parahaemolyticus* planktonic cells, and these effects were dependent on the time of UV exposure.

To date, limited information has been provided in the literature regarding the effectiveness of this technology in more realistic environments where microbial interactions can occur. Thus, the aim of this study was to investigate the impact of microbial interactions on biofilm formation and the resistance of biofilm cells to an innovative photocatalytic surfactant based on TiO_2_ nanoparticles. Thus, various foodborne pathogens (e.g., *Listeria monocytogenes*, *Salmonella enterica*, and *Escherichia coli*) and food spoilage bacteria (e.g., *Pseudomonas fragi*, *Enterobacteriaceae*, *Brochotrix thermosphacta*, *Leuconostoc* spp., and *Latilactobacillus sakei*) were left to form mature biofilms (mono- or dual-species biofilms). These species/strains were selected as representative species that may pose threats of foodborne outbreaks or the spoilage of foods, while the inquiry into their combination was intended to better simulate the environment wherein a pathogen co-exists with other microorganisms. The impact of pathogen–pathogen and pathogen–spoilage bacteria interactions on biofilm formation and resistance to three disinfection strategies, namely, exposure to ultraviolet, an innovative surfactant based on TiO_2_ nanoparticles, and their combination (the photocatalytic application of the surfactant), was examined through a multifactorial experiment incorporating 196 cases (49 microbial combinations × 4 treatments, including a control). This experimental procedure was designed to obtain better insights into the adaptability of food-related microorganisms in industrial environment surfaces and their resistance to disinfectants in vitro, which, hopefully, will provide important data regarding biofilm disinfection within food-processing environments.

## 2. Materials and Methods

### 2.1. Bacterial Strains and Inocula Preparation

The bacterial strains used in the present study to create mono-species and dual-species biofilms have been deposited in the Food Microbiology Culture Collection (FMCC) of the Laboratory of Microbiology and Biotechnology of Foods at the Agricultural University of Athens. Each strain and source of isolation are listed in Table 1. All strains were stored at −80 °C in Tryptic Soy Broth (TSB) (LABM, Bury, UK) containing 20% (*v*/*v*) glycerol (APPLICHEM, Darmstadt, Germany) until further use.

Prior to each experiment, the cultures were activated by adding 10 μL stock culture (−80 °C) to 5 mL of TSB and incubated at temperatures, namely, 25 °C (*Brochothrix thermosphacta* and *Pseudomonas fragi*), 30 °C (*Leuconostoc* spp. and *Latilactobacillus sakei*), and 37 °C (*L. monocytogenes*, *S.* Enteritidis, *E. coli*, *Serratia liquefaciens*, *Serratia proteomaculans*, *Citrobacter freundii*, *Hafnia alvei*, and *Proteus vulgaris*), specifically applied for each groups of microorganisms (pre-culture). After 24 h, 10 μL of each pre-culture was transferred to 10 mL of TSB and incubated under the same conditions for 24 h, 18 h, or 16 h depending on the microorganism (*Pseudomonas*, lactic acid bacteria, and *Br. thermosphacta* or *Enterobacteriaceae*, or pathogens, respectively) in order to prepare the active cultures. To remove any residue of the growth medium, the active culture was centrifuged at 5000× *g* at 4 °C for 10 min (Thermo Fisher Scientific, Waltham, MA, USA). For the single-strain inocula, the cells were washed twice with 10 mL of ¼ strength Ringer solution and finally resuspended in ¼ strength Ringer solution so that they could be used as inocula for the biofilm development assays. To obtain mixed-strain inocula after the end of the second centrifugation procedure, the cells of different strains of each microorganism were mixed. Finally, successive decimal dilutions were made in ¼ strength Ringer sterile solution to create the final mixed-strain-cultured inocula (10^6^ cfu/mL final population).

### 2.2. Biofilm Formation on Stainless-Steel Surface

Attachment of inocula on stainless-steel (SS) coupons (3 × 1 × 0.1 cm) was performed at 15 °C for 3 h after inoculation of bacterial suspensions on 4.5 mL ¼ strength Ringer’s solution (in the case of mono-species cultures) or 4 mL of ¼ strength Ringer’s solution (in the case of mixed-species cultures) according to the method reported by Giaouris et al. [48]. After this step, SS coupons were aseptically transferred into tubes containing 5 mL ¼ strength Ringer’s solution and shaken manually for a few seconds to remove the cells that were loosely attached on SS surface. Finally, the SS coupons were immersed in 5 mL of TSB medium and left to form biofilms at 20 °C for 6 days, for which the medium was renewed after every 48 h.

### 2.3. Disinfection of Stainless-Steel Surface

A ready-to-use titanium-dioxide (TiO_2_)-nanoparticle-based surfactant (Moulas Scientific, Attica, Greece) was used as a disinfectant of the SS coupons. According to the manufacturer, TiO_2_ powder was mixed with acetone (<1%), water, and Triton X-100 (2 drops) under continuous stirring to prepare a final solution of 59.58% (*w*/*v*) (according to the method reported by Tsoukleris et al. [49]). For disinfection procedure, each coupon was rinsed with 5 mL of sterile ¼ strength Ringer solution on each side in order to remove the cells that were loosely attached on SS surface (according to the method reported by Giaouris et al. [48]) and placed into a sterile petri dish. Prior to the application of UV irradiation, a volume of 500 μL TiO_2_ solution was spread with the use of a pipette on SS coupons’ surface. After 2 h of exposure to UV, the SS coupons were inverted, and the same procedure was followed to disinfect the other side of the SS coupon. In the case of control samples, the biofilm population of each microbial combination was estimated after removing the loosely attached cells [48] without any further treatment. Additionally, UV-treated samples, i.e., SS coupons exposed to UV treatment without the addition of TiO_2_ solution, and TiO_2_-treated samples, i.e., the coupons treated with TiO_2_ without exposure to UV, were also included in this study.

### 2.4. Quantitation of Viable Biofilm Cells Using the Bead-Vortexing Method

Determination of biofilm populations formed on the surface of SS coupons was performed before (control samples) and after disinfection according to the bead-votexing method [50,51]. The media PALCAM Agar (Palcam, Biokar Diagnostics, Allonne, France, with selective supplement BS00408); TBX (OXOID, Hampshire, UK); Xylose Lysine Deoxycholate (XLD, Oxoid); Streptomycin Thallous Acetate-Actidione Agar (STAA, Biolife, Milano, Italy); VRBGA (VRBGA, OXOID, Hampshire, UK); De Man, Rogosa, and Sharpe agar (MRS ISO, LABM, Bury, UK); and *Pseudomonas* Agar Base with selective supplement (PAB, Biolife, Milano, Italy) were used for the enumeration of *L. monocytogenes*, *E. coli*, *S. enteritidis*, *Br. thermosphacta*, *Enterobacteriaceae*, lactic acid bacteria (LAB), and *Ps. fragi*, respectively. The selectivity of the used growth media was examined via inoculation of the bacterial species tested in this study. The results were expressed (log CFU/cm^2^ ± SD) as mean values of the six replicates (at least), which were performed per case.

### 2.5. Statistical Analysis and Data Visualization

**Figure 1:**
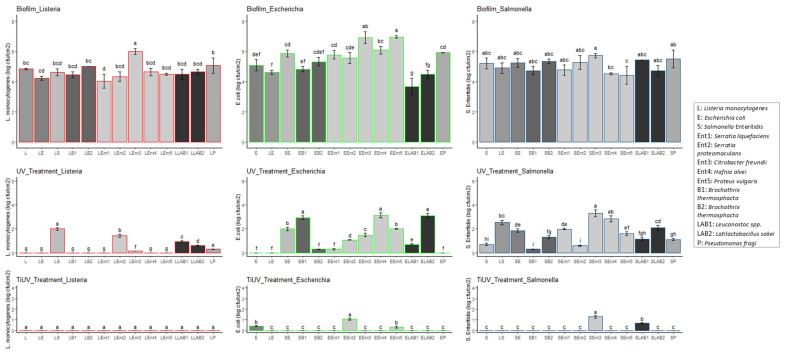
Dual-strain and dual-species biofilm populations (log cfu/cm^2^) of *Listeria monocytogenes* (Red), *Escherichia coli* (Green), and *Salmonella* Enteritidis (Βlue) without treatment (Biofilm) (Control), after exposure to UV for 2 h (UV_Treatment) and to the photocatalytic TiO_2_-nanoparticle-based surfactant (TiO_2_ plus UV for 2 h—TiUV_Treatment). The error bars represent the standard deviation of six replicates. The different letters (i.e., a, b, c, d, e, f, g, h, and i) represent the significant differences (*p* < 0.05) between the population of each pathogen under mono-species culture and different microbial combination cultures (dual-species) based on the specified tested condition (Biofilm or UV_Treatment or TiUV_Treatment).

**Figure 2:**
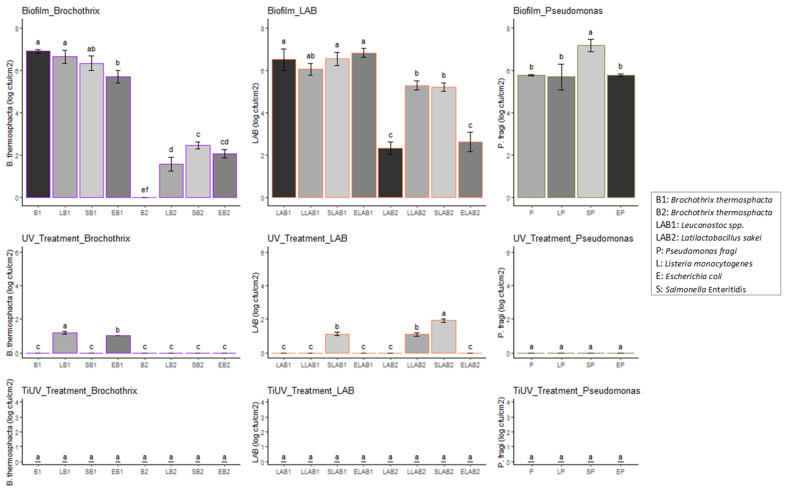
Mono- and dual-species biofilm populations (log cfu/cm^2^) of *Brochothrix thermosphacta* (purple), lactic acid bacteria (LAB) (coral), and *Pseudomonas fragi* (olivegreen) without treatment (Biofilm) (Control), after exposure to UV for 2 h (UV_Treatment) and to the photocatalytic TiO_2_-nanoparticle-based surfactant (TiO_2_ plus UV for 2 h—TiUV_Treatment). The error bars represent the standard deviation of six replicates. The different letters (i.e., a, b, c, and d) represent the significant differences (*p* < 0.05) between the population of each species under mono-species culture and different microbial combination cultures (dual-species) based on the specified tested condition (Biofilm or UV_Treatment or TiUV_Treatment).

**Figure 3:**
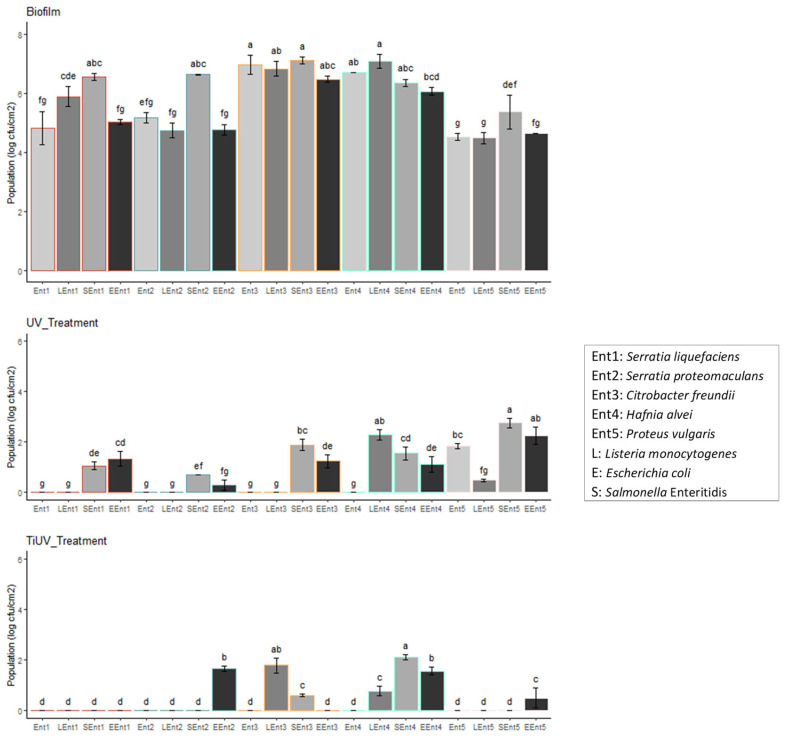
Mono- and dual-species biofilm populations (log cfu/cm^2^) of *Enterobacteriaceae* without treatment (Biofilm) (Control), after exposure to UV for 2 h (UV_Treatment) and to the photocatalytic TiO_2_-nanoparticle-based surfactant (TiO_2_ plus UV for 2 h—TiUV_Treatment). The error bars represent the standard deviation of six replicates. The different letters (i.e., a, b, c, d, e, f, and g) represent the significant differences (*p* < 0.05) between the population of each microbe under mono-species culture and different microbial combination cultures (dual-species) based on the specified tested condition (Biofilm or UV_Treatment or TiUV_Treatment).

A total of 196 different cases were tested in this study by means of the exposure of 49 microbial combinations to 4 treatments (including a control). One- and two-way ANOVA tests were performed to estimate the significant differences (*p* < 0.05) between the populations of the different microbes under mono- and dual-species biofilms of each tested condition i.e., biofilm formation (Biofilm), exposure to TiO_2_-nanoparticle-based surfactant (TiO_2_ for 2 h—TiO_2__Treatment), exposure to UV for 2 h (UV_Treatment), or exposure to the photocatalytic TiO_2_-nanoparticle-based surfactant (TiO_2_ plus UV for 2 h—TiUV_Treatment). The packages ggplot2, ggpubr, tidyverse, broom, AICcmodavg, and multcompView of R studio (2021.09.2 + 382) were used for the statistical analysis and visualization of the results. The significant differences between the population of each pathogen or spoilage microbial species under mono-species culturing and in different microbial combinations, i.e., dual-species cultures based on the specified tested condition (Biofilm, UV_Treatment, or TiUV_Treatment), were estimated and represented by different letters (i.e., a, b, c, d, e, f, g, h, and i) in Figure 1, Figure 2 and Figure 3.

## 3. Results and Discussion

The presence of microorganisms on food-processing surfaces and environments poses the risk of the contamination of products, which may lead to reductions in the shelf-life of products, food losses, and the presence of spoilage and/or pathogenic microorganisms in food [52,53]. It has been reported that different factors alone or synergistically affect the adherence of cells to and the formation of biofilms on a surface, including the surfaces’ material, the temperature, the availability of nutrients, and the presence of other microbial species [8,54,55,56,57,58]. The ability of microorganisms to form biofilms in these environments and the difficulties that may arise in removing these communities from industrial surfaces [27,59] are of great importance for the food industry. In the present study, the influence of the microbial interactions of pathogenic and spoilage bacteria on the population of biofilms and their decontamination by an innovative, TiO_2_-nanoparticle-based surfactant was evaluated. The species examined in this study (Table 1) were chosen as they represent commonly detected pathogenic and spoilage bacteria in industrial food ecosystems. These microbial species were left to form mature biofilms (6 days) on stainless-steel surfaces at 20 °C to better stimulate the conditions in an industrial environment, especially in cases where a surface is not properly disinfected. The occurrence of the latter concern can be attributed to the difficulty of reaching several spots with the disinfection procedure applied and the presence of scratches, fissures, and angles that enable the trapping of microorganisms [60].

Figure 1, Figure 2 and Figure 3 illustrate that all the microorganisms were able to form mature biofilms under mono- or dual-species conditions. Numerous studies have been conducted to describe the ability of pathogenic microorganisms, including *L. monocytogenes*, *E. coli*, and *Salmonella*, to adhere to food-processing surfaces [54,61,62,63,64,65,66,67]. According to the present study, the biofilm population enumerated on stainless steel surfaces was dependent on the strain or species left to form the biofilm on mono-species or dual species biofilm (*p* < 0.05). In Figure 1, it is shown that the *L. monocytogenes* dual-strain biofilm population was affected by the presence of *Serratia liquefaciens* and *Citrobacter freundii*. In the case of the *E. coli* biofilm, the presence of most of the *Enterobacteriaceae* and both strains of LAB was observed to affect the *E. coli* population (*p* < 0.05). The influence of the pathogens on the formation of biofilms by the rest of the tested microorganisms was also shown in this study. In brief, *L. monocytogenes* affected the biofilm population of *Br. thermosphacta* strain B-434, *Ltb*. *sakei*, and *Ser. liquefaciens*. Similarly, *E. coli* influenced the biofilm cell density of *Br. thermosphacta* strains. Moreover, the *Br. thermosphacta* B-434, *Ltb. sakei*, *Ps. fragi*, *Serratia* species, and *Proteus vulgaris* biofilm populations were affected by the presence of *Salmonella* Enteritidis. Interestingly enough, the biofilm population of *Salmonella* Enteritidis was not affected by the presence of other species (Figure 1). Similar results have been previously reported regarding the inability of *Listeria* to affect the biofilm population of *Salmonella* and vice versa [50], and no cause of bulk environmental samples on *Salmonella* biofilm cells [68]. The observation that microbial interactions may affect (increase or reduce) or fail to affect the biofilm formation of a specific microorganism has been reported previously [32,33,69,70,71,72,73,74]. Moreover, the role of microbial interactions in biofilms formed by food-related microorganisms has been shown [75,76,77,78]. Particularly, the biofilm cells of those pathogenic species were found to be reduced by the presence of *Lactococcus lactis*, *Ltb. sakei*, and *Latilactobacillus curvatus* [79], while *Lactococcus lactis* or *Flavobacterium* spp. affected the adhesion ability of *L. monocytogenes* [69,70]. In a recent study, a pseudomonads population was found to increase the cell density of *Listeria* biofilms [80], while the biofilm cell density of various *Pseudomonas* strains was increased when co-cultured with *E. coli* and differentially affected by the presence of *Salmonella* Typhimurium [81]. Furthermore, Habimana et al. [82] found that the presence of *Acinetobacter calcoaceticus* enhanced the biofilm capacity of *E. coli*. The observed inability of *H. alvei* to affect the biofilm formation of *Salmonella* has been reported previously [83].

Regarding the disinfection procedure, the resistance to all different disinfection treatments was dependent on the different tested microbial combinations (*p* < 0.05). The disinfection of biofilms with TiO_2_ for 2 h did not reduce the biofilm populations significantly (below the detection limit of the method); however, the remaining population was affected by the microbial combinations in most of the cases. On the contrary, exposure to UV reduced the populations of the biofilm cells in most cases, reducing such populations below the detection limit of the method in some cases. Moreover, the use of the disinfectant strengthened the antimicrobial activity of UV irradiation (Figure 1, Figure 2 and Figure 3). In brief, the exposure of biofilm communities for 2 h to TiO_2_ and UV was found to be an effective decontamination procedure in most cases (Figure 1 and Figure 3). The effectiveness of the use of TiO_2_ nanoparticles for microbial inactivation has also been previously reported [37,39,40,41,42,84,85,86,87,88,89,90,91,92,93]. The results of the present work complement the observations of a previous research study wherein the combined use of UV irradiation with TiO_2_ nanoparticles was found to be a promising procedure for the decontamination of biofilms formed by *Listeria* [84], as various pathogenic and spoilage bacteria were tested. It seems that the examined photocatalytic TiO_2_-nanoparticle-based surfactant could be a promising disinfectant with which to ensure not only food safety but also food quality by preventing its contamination by spoilage bacteria. It must be noted that the decontamination activity of the examined surfactant could be attributed to the photocatalysis of TiO_2_, as the exposure only to TiO_2_ for 2 h did not reduce the biofilm populations significantly. In addition, it could be hypothesized that the low concentration of the rest of the ingredients of the surfactant, i.e., acetone and Triton X-100, was not able to present or presented only weak antibacterial activity. The weak antimicrobial activity of the latter toward *Bacillus* and *Escherichia* has been observed before [94].

The increased resistance of species to different disinfectants as a result of the presence of other microorganisms [32,48,50,95,96,97] has to be taken into account. In the present study, the presence of *Salmonella* Enteritidis, *Serratia proteomaculans*, *C. freundii*, *Leuconostoc* spp., *Ltb. sakei,* and *Ps. fragi* was found to increase the resistance of *Listeria* biofilm cells to UV disinfection for 2 h, but none of the microbial combinations were found to affect the resistance of *Listeria* biofilm cells to exposure to TiO_2_ and UV for 2 h (Figure 1). A higher sensitivity of the single-species biofilms of the *L. monocytogenes* and *L. plantarum* cultures to disinfectants compared to the dual-species biofilm was mentioned before [32]. In another study, the resistance of *L. monocytogenes* to benzalkonium chloride was not affected by the presence of *Pseudomonas putida* cells [48]. In this study, the effect of microbial interactions on the resistance to the UV treatment (*p* < 0.05) of the *E. coli* biofilm was more obvious than on the effect induced by TiO_2_ and UV exposure for 2 h (*p* < 0.05). Moreover, the resistance of the *Salmonella* biofilm cells to UV treatment was affected by the presence of most of the tested species, while resistance to TiO_2_ and UV exposure for 2 h was affected by two species (*C. freundii* and *Leuconostoc* spp.) (Figure 1). In contrast to these results, the *Listeria*–*Salmonella* interactions did not influence the antimicrobial resistance of either species [50]. In Figure 2 and Figure 3, the effects of *L. monocytogenes* on the resistance of *Br. thermosphacta* strain B-432, *Ltb. sakei*, *H. alvei*, and *Proteus vulgaris* to UV exposure are shown. In brief, *L. monocytogenes* was found to increase the resistance of these microorganisms to UV exposure, although no significant differences were observed regarding the resistance of the rest of the tested microorganisms influenced by the presence of *Listeria* cells (Figure 2 and Figure 3). In a previous study, an increased level of resistance of *Ps. putida* to benzalkonium chloride was observed in a dual-species biofilm with *L. monocytogenes* [48]; however, the increased resistance *Ps. fragi* cells to different treatments in a dual-species biofilm was not observed in this study (Figure 2). In addition, *E. coli* influenced the resistance of *Br. thermosphacta* strain B-432, *Ser. liquefaciens*, *Ser. proteomaculans*, *C. freundii*, and *H. alvei* to UV exposure (Figure 2 and Figure 3). Similarly, an increased level of resistance of LAB and all spoilage *Enterobacteriaceae* to UV treatment was observed in the dual-species biofilm with *Salmonella* (Figure 2 and Figure 3). No biofilm cells of *Brochothrix*, LAB, *Ps. fragi*, or *Enterobacteriaceae* were enumerated after the single-species communities’ exposure to TiO_2_ and UV for 2 h (Figure 2 and Figure 3). Similarly, the presence of the three pathogens did not affect the resistance of *Brochothrix*, LAB, or *Ps. fragi* to TiO_2_ and UV after 2 h of exposure (Figure 2). However, *L. monocytogenes* was found to increase the resistance of *C. freundii* and *H. alvei* to exposure to TiO_2_ and UV for 2 h (Figure 3). Interestingly enough, *Salmonella* also increased the resistance of two strains, namely, *C. freundii* and *H. alvei*, to TiO_2_ and UV for the 2 h treatment (Figure 3). Moreover, the presence of *E. coli* cells was found to affect the resistance of *Ser. Proteomaculans*, *H. alvei*, and *Proteus vulgaris* to the TiO_2_ and UV treatments (Figure 3). In another study, the sensitivity of *Proteus vulgaris*, *E. coli*, and *Pseudomonas aeruginosa*, *Streptococcus pneumonia*, and *Staphylococcus aureus* to synthetic TiO_2_ nanoparticles was estimated [98]. The observation made by Fu et al. [99], wherein the sensitivity of Gram-positive microorganisms to exposure to TiO_2_ is more pronounced than in Gram-negative ones, was also confirmed in this study, as biofilm populations were only enumerated for *Enterobacteriaceae* members (pathogenic or non-pathogenic species) after treatment with TiO_2_ and UV for 2 h (Figure 1, Figure 2 and Figure 3). However, opposite results were reported in a recent study, wherein the antibiofilm activity of green-synthesized TiO_2_ nanoparticles was found to be more effective in *C. freundii* than in *Streptococcus* cells [100]. Moreover, the higher resistance of *Listeria* planktonic cells to a TiO_2_ photocatalyst compared to *Salmonella* cells [40] was not confirmed in this research.

## 4. Conclusions

From all the above, it seems that microbial species prefer to gather together on a surface than be found free in a natural environment. Moreover, it was highlighted once again that the presence of more than one species affected both cells’ biofilm formation ability and their resistance to ultraviolet radiation and a disinfectant. This information is of great importance, as mixed communities are present in the environment. In addition, the observation that the UV treatment was insufficient in terms of sanitizing the surfaces where a mature biofilm had been formed by pathogens, especially in mixed communities, must be taken into account. However, in this study, it was shown that the examined agent, i.e., the photocatalytic TiO_2_-nanoparticle-based surfactant, enhanced the effectiveness of UV with respect to the disinfection of biofilms formed by various microorganisms, suggesting that this could represent an alternative way of disinfecting contaminated surfaces. Further studies are needed to explore this phenomenon in depth (including gene expression studies, an analysis including real food or the food industry, etc.) as these results present an intriguing case that may provide powerful solutions regarding biofilm disinfection within food-processing environments.

## Figures and Tables

**Table 1:** Pathogenic and spoilage bacterial strains used in the present study.

Species	FMCC CODE *	Strain	Sources
*Listeria monocytogenes*	B-129	21350	Frozen meal (meat-based)
*Listeria monocytogenes* *E. coli* O157:H7	B-128	21085	Soft Cheese
	B-18	NCTC 13127	Human feces
*E. coli* O157:H7 *Salmonella enterica ser.* Enteritidis	B-289	ATCC 35150	Human feces
	B-56	WT	Provided by Prof L. Cocolin
*Salmonella enterica ser.* Enteritidis	B-287	P167807	Provided by Surrey University
*Brochothrix thermosphacta*	Β-432	20A3	Pork [45]
*Brochothrix thermosphacta* *Serratia liquefaciens*	B-434	4A1	Pork [45] Minced beef [46]
	Β-292	VK6	
*Serratia proteomaculans*	Β-293	VK17	Minced beef [46]
*Citrobacter freundii*	Β-294	VK19	Minced beef [46]
*Hafnia alvei*	Β-295	VK20	Minced beef [46]
*Proteus vulgaris*	Β-306	VK101	Minced beef [46]
*Leuconostoc* spp.	Β-233	-	Minced beef [47]
*Latilactobacillus sakei*	Β-226	-	Minced beef [47]
*Pseudomonas fragi*	Β-209	DSM–3456	Unknown

* FMCC: Food Microbiology Culture Collection, Laboratory of Microbiology and Biotechnology of Foods, Agricultural University of Athens.

## Data Availability

Data is contained within the article.

## References

[1] Henrici A.T. (1933). Studies of Freshwater Bacteria. J. Bacteriol..

[2] Zobell C.E., Allen E.C. (1935). The Significance of Marine Bacteria in the Fouling of Submerged Surfaces. J. Bacteriol..

[3] Høiby N. (2017). A Short History of Microbial Biofilms and Biofilm Infections. APMIS.

[4] Donlan R.M., Costerton J.W. (2002). Biofilms: Survival Mechanisms of Clinically Relevant Microorganisms. Clin. Microbiol. Rev..

[5] Sauer K., Rickard A., Davies D. (2007). Biofilms and Biocomplexity. Microbe Wash. DC.

[6] Subramani R., Jayaprakashvel M., Bramhachari P.V. (2019). Bacterial Quorum Sensing: Biofilm Formation, Survival Behaviour and Antibiotic Resistance. Implication of Quorum Sensing and Biofilm Formation in Medicine, Agriculture and Food Industry.

[7] Costerton J.W., Irvin R.T., Cheng K.J. (1981). The Role of Bacterial Surface Structures in Pathogenesis. Crit. Rev. Microbiol..

[8] Costerton J.W., Cheng K.J., Geesey G.G., Ladd T.I., Nickel J.C., Dasgupta M., Marrie T.J. (1987). Bacterial Biofilms in Nature and Disease. Annu. Rev. Microbiol..

[9] McEldowney S., Fletcher M. (1988). Bacterial Desorption from Food Container and Food Processing Surfaces. Microb. Ecol..

[10] James G.A., Beaudette L., Costerton J.W. (1995). Interspecies Bacterial Interactions in Biofilms. J. Ind. Microbiol..

[11] Azeredo J., Azevedo N.F., Briandet R., Cerca N., Coenye T., Costa A.R., Desvaux M., Di Bonaventura G., Hébraud M., Jaglic Z. (2017). Critical Review on Biofilm Methods. Crit. Rev. Microbiol..

[12] Fletcher M., Beachey E.H. (1980). Adherence of Marine Micro-Organisms to Smooth Surfaces. Bacterial Adherence.

[13] Costerton J.W., Gessey G.G., Mittal K.L. (1979). Microbial Contamination of Surfaces. Surface Contamination: Genesis, Detection, and Control.

[14] Jones H.C., Roth I.L., Sanders W.M. (1969). Electron Microscopic Study of a Slime Layer. J. Bacteriol..

[15] Bassler B.L. (1999). How Bacteria Talk to Each Other: Regulation of Gene Expression by Quorum Sensing. Curr. Opin. Microbiol..

[16] Falà A.K., Álvarez-Ordóñez A., Filloux A., Gahan C.G.M., Cotter P.D. (2022). Quorum Sensing in Human Gut and Food Microbiomes: Significance and Potential for Therapeutic Targeting. Front. Microbiol..

[17] Giaouris E., Chorianopoulos N., Skandamis P., Nychas G.J. (2012). Attachment and Biofilm Formation by *Salmonella* in Food Processing Environments. Salmonella: A Dangerous Foodborne Pathogen.

[18] Dalton H.M., Goodman A.E., Marshall K.C. (1996). Diversity in Surface Colonization Behavior in Marine Bacteria. J. Ind. Microbiol. Biotechnol..

[19] Shapiro J.A., Hsu C. (1989). *Escherichia Coli* K-12 Cell-Cell Interactions Seen by Time-Lapse Video. J. Bacteriol..

[20] Swift S., Bainton N.J., Winson M.K. (1994). Gram-Negative Bacterial Communication by N-Acyl Homoserine Lactones: A Universal Language?. Trends Microbiol..

[21] Marshall K.C., Alexander M. (1960). Competition between Soil Bacteria and Fusarium. Plant Soil.

[22] Barnes L.-M., Lo M.F., Adams M.R., Chamberlain A.H.L. (1999). Effect of Milk Proteins on Adhesion of Bacteria to Stainless Steel Surfaces. Appl. Environ. Microbiol..

[23] Kusumaningrum H.D., Riboldi G., Hazeleger W.C., Beumer R.R. (2003). Survival of Foodborne Pathogens on Stainless Steel Surfaces and Cross-Contamination to Foods. Int. J. Food Microbiol..

[24] Giaouris E., Chorianopoulos N., Nychas G.-J.E. (2005). Effect of Temperature, PH, and Water Activity on Biofilm Formation by *Salmonella enterica* Enteritidis PT4 on Stainless Steel Surfaces as Indicated by the Bead Vortexing Method and Conductance Measurements. J. Food Prot..

[25] Brandl M.T. (2006). Fitness of Human Enteric Pathogens on Plants and Implications for Food Safety. Annu. Rev. Phytopathol..

[26] Murphy C., Carroll C., Jordan K.N. (2006). Environmental Survival Mechanisms of the Foodborne Pathogen *Campylobacter jejuni*. J. Appl. Microbiol..

[27] Gandhi M., Chikindas M.L. (2007). *Listeria*: A Foodborne Pathogen That Knows How to Survive. Int. J. Food Microbiol..

[28] Dunsmore D.G. (1981). Bacteriological Control of Food Equipment Surfaces by Cleaning Systems. I. Detergent Effects. J. Food Prot..

[29] Sharma M., Anand S.K. (2002). Characterization of Constitutive Microflora of Biofilms in Dairy Processing Lines. Food Microbiol..

[30] Srinivasan S., Harrington G.W., Xagoraraki I., Goel R. (2008). Factors Affecting Bulk to Total Bacteria Ratio in Drinking Water Distribution Systems. Water Res..

[31] Yuan L., Sadiq F.A., Wang N., Yang Z., He G. (2021). Recent Advances in Understanding the Control of Disinfectant-Resistant Biofilms by Hurdle Technology in the Food Industry. Crit. Rev. Food Sci. Nutr..

[32] van der Veen S., Abee T. (2011). Mixed Species Biofilms of *Listeria monocytogenes* and *Lactobacillus Plantarum* Show Enhanced Resistance to Benzalkonium Chloride and Peracetic Acid. Int. J. Food Microbiol..

[33] Giaouris E., Heir E., Hébraud M., Chorianopoulos N., Langsrud S., Møretrø T., Habimana O., Desvaux M., Renier S., Nychas G.-J. (2014). Attachment and Biofilm Formation by Foodborne Bacteria in Meat Processing Environments: Causes, Implications, Role of Bacterial Interactions and Control by Alternative Novel Methods. Meat Sci..

[34] Morones J.R., Elechiguerra J.L., Camacho A., Holt K., Kouri J.B., Ramírez J.T., Yacaman M.J. (2005). The Bactericidal Effect of Silver Nanoparticles. Nanotechnology.

[35] Pal S., Tak Y.K., Song J.M. (2007). Does the Antibacterial Activity of Silver Nanoparticles Depend on the Shape of the Nanoparticle? A Study of the Gram-Negative Bacterium *Escherichia coli*. Appl. Environ. Microbiol..

[36] El Badawy A.M., Silva R.G., Morris B., Scheckel K.G., Suidan M.T., Tolaymat T.M. (2011). Surface Charge-Dependent Toxicity of Silver Nanoparticles. Environ. Sci. Technol..

[37] Fujishima A., Hashimoto K., Watanabe T. (1999). TiO_2_ Photocatalysis: Fundamentals and Applications.

[38] Fujishima A., Rao T.N., Tryk D.A. (2000). Titanium Dioxide Photocatalysis. J. Photochem. Photobiol. C Photochem. Rev..

[39] Matsunaga T., Tomoda R., Nakajima T., Nakamura N., Komine T. (1988). Continuous-Sterilization System That Uses Photosemiconductor Powders. Appl. Environ. Microbiol..

[40] Kim B., Kim D., Cho D., Cho S. (2003). Bactericidal Effect of TiO_2_ Photocatalyst on Selected Food-Borne Pathogenic Bacteria. Chemosphere.

[41] Maneerat C., Hayata Y. (2006). Antifungal Activity of TiO_2_ Photocatalysis against *Penicillium Expansum* in Vitro and in Fruit Tests. Int. J. Food Microbiol..

[42] Duffy E.F., Al Touati F., Kehoe S.C., McLoughlin O.A., Gill L.W., Gernjak W., Oller I., Maldonado M.I., Malato S., Cassidy J. (2004). A Novel TiO_2_-Assisted Solar Photocatalytic Batch-Process Disinfection Reactor for the Treatment of Biological and Chemical Contaminants in Domestic Drinking Water in Developing Countries. Sol. Energy.

[43] Fujishima A., Honda K. (1972). Electrochemical Photolysis of Water at a Semiconductor Electrode. Nature.

[44] Ireland J.C., Klostermann P., Rice E.W., Clark R.M. (1993). Inactivation of Escherichia Coli by Titanium Dioxide Photocatalytic Oxidation. Appl. Environ. Microbiol..

[45] Papadopoulou O.S., Doulgeraki A.I., Botta C., Cocolin L., Nychas G.-J.E. (2012). Genotypic Characterization of *Brochothrix Thermosphacta* Isolated during Storage of Minced Pork under Aerobic or Modified Atmosphere Packaging Conditions. Meat Sci..

[46] Doulgeraki A.I., Paramithiotis S., Nychas G.-J.E. (2011). Characterization of the *Enterobacteriaceae* Community That Developed during Storage of Minced Beef under Aerobic or Modified Atmosphere Packaging Conditions. Int. J. Food Microbiol..

[47] Doulgeraki A.I., Paramithiotis S., Kagkli D.M., Nychas G.-J.E. (2010). Lactic Acid Bacteria Population Dynamics during Minced Beef Storage under Aerobic or Modified Atmosphere Packaging Conditions. Food Microbiol..

[48] Giaouris E., Chorianopoulos N., Doulgeraki A., Nychas G.-J. (2013). Co-Culture with *Listeria Monocytogenes* within a Dual-Species Biofilm Community Strongly Increases Resistance of *Pseudomonas Putida* to Benzalkonium Chloride. PLoS ONE.

[49] Tsoukleris D.S., Maggos T., Vassilakos C., Falaras P. (2007). Photocatalytic Degradation of Volatile Organics on TiO_2_ Embedded Glass Spherules. Catal. Today.

[50] Kostaki M., Chorianopoulos N., Braxou E., Nychas G.-J., Giaouris E. (2012). Differential Biofilm Formation and Chemical Disinfection Resistance of Sessile Cells of *Listeria Monocytogenes* Strains under Monospecies and Dual-Species (with *Salmonella enterica*) Conditions. Appl. Environ. Microbiol..

[51] Chorianopoulos N.G., Giaouris E.D., Skandamis P.N., Haroutounian S.A., Nychas G.-J.E. (2008). Disinfectant Test against Monoculture and Mixed-Culture Biofilms Composed of Technological, Spoilage and Pathogenic Bacteria: Bactericidal Effect of Essential Oil and Hydrosol of *Satureja Thymbra* and Comparison with Standard Acid–Base Sanitizers. J. Appl. Microbiol..

[52] Hall-Stoodley L., Costerton J.W., Stoodley P. (2004). Bacterial Biofilms: From the Natural Environment to Infectious Diseases. Nat. Rev. Microbiol..

[53] Jessen B., Lammert L. (2003). Biofilm and Disinfection in Meat Processing Plants. Int. Biodeterior. Biodegrad..

[54] Chmielewski R.A.N., Frank J.F. (2003). Biofilm Formation and Control in Food Processing Facilities. Compr. Rev. Food Sci. Food Saf..

[55] Rivas L., Dykes G.A., Fegan N. (2007). A Comparative Study of Biofilm Formation by Shiga Toxigenic *Escherichia Coli* Using Epifluorescence Microscopy on Stainless Steel and a Microtitre Plate Method. J. Microbiol. Methods.

[56] Rode T.M., Langsrud S., Holck A., Møretrø T. (2007). Different Patterns of Biofilm Formation in *Staphylococcus Aureus* under Food-Related Stress Conditions. Int. J. Food Microbiol..

[57] Van Houdt R., Michiels C.W. (2010). Biofilm Formation and the Food Industry, a Focus on the Bacterial Outer Surface. J. Appl. Microbiol..

[58] Herrera J.J.R., Cabo M.L., González A., Pazos I., Pastoriza L. (2007). Adhesion and Detachment Kinetics of Several Strains of *Staphylococcus Aureus* Subsp. *Aureus* under Three Different Experimental Conditions. Food Microbiol..

[59] Gilbert M.T.P., Hansen A.J., Willerslev E., Rudbeck L., Barnes I., Lynnerup N., Cooper A. (2003). Characterization of Genetic Miscoding Lesions Caused by Postmortem Damage. Am. J. Hum. Genet..

[60] Kumar C.G., Anand S.K. (1998). Significance of Microbial Biofilms in Food Industry: A Review. Int. J. Food Microbiol..

[61] Mai T.L., Conner D.E. (2007). Effect of Temperature and Growth Media on the Attachment of *Listeria Monocytogenes* to Stainless Steel. Int. J. Food Microbiol..

[62] Frank J.F., Koffi R.A. (1990). Surface-Adherent Growth of *Listeria Monocytogenes* Is Associated with Increased Resistance to Surfactant Sanitizers and Heat. J. Food Prot..

[63] Herald P.J., Zottola E.A. (1988). Attachment of *Listeria Monocytogenes* to Stainless Steel Surfaces at Various Temperatures and PH Values. J. Food Sci..

[64] Mafu A.A., Roy D., Goulet J., Magny P. (1990). Attachment of *Listeria monocytogenes* to Stainless Steel, Glass, Polypropylene, and Rubber Surfaces After Short Contact Times. J. Food Prot..

[65] Dewanti R., Wong A.C.L. (1995). Influence of Culture Conditions on Biofilm Formation by *Escherichia Coli* O157:H7. Int. J. Food Microbiol..

[66] Joseph B., Otta S.K., Karunasagar I., Karunasagar I. (2001). Biofilm Formation by *Salmonella* Spp. on Food Contact Surfaces and Their Sensitivity to Sanitizers. Int. J. Food Microbiol..

[67] Kadam S.R., den Besten H.M.W., van der Veen S., Zwietering M.H., Moezelaar R., Abee T. (2013). Diversity Assessment of *Listeria monocytogenes* Biofilm Formation: Impact of Growth Condition, Serotype and Strain Origin. Int. J. Food Microbiol..

[68] Doulgeraki A.I., Papaioannou M., Nychas G.-J.E. (2016). Targeted Gene Expression Study of *Salmonella enterica* during Biofilm Formation on Rocket Leaves. LWT-Food Sci. Technol..

[69] Bremer P.J., Monk I., Osborne C.M. (2001). Survival of *Listeria monocytogenes* Attached to Stainless Steel Surfaces in the Presence or Absence of *Flavobacterium* spp.. J. Food Prot..

[70] Habimana O., Meyrand M., Meylheuc T., Kulakauskas S., Briandet R. (2009). Genetic Features of Resident Biofilms Determine Attachment of *Listeria monocytogenes*. Appl. Environ. Microbiol..

[71] Zijnge V., van Leeuwen M.B.M., Degener J.E., Abbas F., Thurnheer T., Gmür R., Harmsen H.J.M. (2010). Oral Biofilm Architecture on Natural Teeth. PLoS ONE.

[72] Lyautey E., Lacoste B., Ten-Hage L., Rols J.-L., Garabetian F. (2005). Analysis of Bacterial Diversity in River Biofilms Using 16S RDNA PCR-DGGE: Methodological Settings and Fingerprints Interpretation. Water Res..

[73] Simões M., Simões L.C., Pereira M.O., Vieira M.J. (2008). Antagonism between *Bacillus cereus* and *Pseudomonas fluorescens* in Planktonic Systems and in Biofilms. Biofouling.

[74] Burmølle M., Thomsen T.R., Fazli M., Dige I., Christensen L., Homøe P., Tvede M., Nyvad B., Tolker-Nielsen T., Givskov M. (2010). Biofilms in Chronic Infections—A Matter of Opportunity—Monospecies Biofilms in Multispecies Infections. FEMS Immunol. Med. Microbiol..

[75] Zhou L., Zhang Y., Ge Y., Zhu X., Pan J. (2020). Regulatory Mechanisms and Promising Applications of Quorum Sensing-Inhibiting Agents in Control of Bacterial Biofilm Formation. Front. Microbiol..

[76] Machado M.A.A., Ribeiro W.A., Toledo V.S., Ramos G.L.P.A., Vigoder H.C., Nascimento J.S. (2020). Antibiotic Resistance and Biofilm Production in Catalase-Positive Gram-Positive Cocci Isolated from Brazilian Pasteurized Milk. J. Food Qual. Hazards Control.

[77] Skandamis P.N., Nychas G.-J.E. (2012). Quorum Sensing in the Context of Food Microbiology. Appl. Environ. Microbiol..

[78] Giaouris E., Heir E., Desvaux M., Hébraud M., Møretrø T., Langsrud S., Doulgeraki A., Nychas G.-J., Kačániová M., Czaczyk K. (2015). Intra- and Inter-Species Interactions within Biofilms of Important Foodborne Bacterial Pathogens. Front. Microbiol..

[79] Gomez G.F., Huang R., MacPherson M., Ferreira Zandona A.G., Gregory R.L. (2016). Photo Inactivation of *Streptococcus* Mutans Biofilm by Violet-Blue Light. Curr. Microbiol..

[80] Papaioannou E., Giaouris E.D., Berillis P., Boziaris I.S. (2018). Dynamics of Biofilm Formation by *Listeria monocytogenes* on Stainless Steel under Mono-Species and Mixed-Culture Simulated Fish Processing Conditions and Chemical Disinfection Challenges. Int. J. Food Microbiol..

[81] Zarei M., Bahrami S., Liljebjelke K. (2022). Biofilm Formation of *Salmonella enterica* Serovar Enteritidis Cocultured with Acanthamoeba Castellanii Responds to Nutrient Availability. Int. Microbiol..

[82] Habimana O., Møretrø T., Langsrud S., Vestby L.K., Nesse L.L., Heir E. (2010). Micro Ecosystems from Feed Industry Surfaces: A Survival and Biofilm Study of *Salmonella* versus Host Resident Flora Strains. BMC Vet. Res..

[83] Blana V., Georgomanou A., Giaouris E. (2017). Assessing Biofilm Formation by *Salmonella enterica* Serovar Typhimurium on Abiotic Substrata in the Presence of Quorum Sensing Signals Produced by *Hafnia alvei*. Food Control.

[84] Chorianopoulos N., Giaouris E., Grigoraki I., Skandamis P., Nychas G.-J. (2011). Effect of Acid Tolerance Response (ATR) on Attachment of *Listeria monocytogenes* Scott A to Stainless Steel under Extended Exposure to Acid or/and Salt Stress and Resistance of Sessile Cells to Subsequent Strong Acid Challenge. Int. J. Food Microbiol..

[85] Cho M., Chung H., Choi W., Yoon J. (2004). Linear Correlation between Inactivation of *E. Coli* and OH Radical Concentration in TiO_2_ Photocatalytic Disinfection. Water Res..

[86] Horie Y., Taya M., Tone S. (1998). Effect of Cell Adsorption on Photosterilization of *Escherichia Coli* over Titanium Dioxide-Activated Charcoal Granules. J. Chem. Eng. Jpn..

[87] Hur J.-S., Oh S.-O., Lim K.-M., Jung J.S., Kim J.-W., Koh Y.J. (2005). Novel Effects of TiO_2_ Photocatalytic Ozonation on Control of Postharvest Fungal Spoilage of Kiwifruit. Postharvest Biol. Technol..

[88] Kikuchi Y., Sunada K., Iyoda T., Hashimoto K., Fujishima A. (1997). Photocatalytic Bactericidal Effect of TiO_2_ Thin Films: Dynamic View of the Active Oxygen Species Responsible for the Effect. J. Photochem. Photobiol. Chem..

[89] Maness P.-C., Smolinski S., Blake D.M., Huang Z., Wolfrum E.J., Jacoby W.A. (1999). Bactericidal Activity of Photocatalytic TiO_2_ Reaction: Toward an Understanding of Its Killing Mechanism. Appl. Environ. Microbiol..

[90] Matsunaga T., Tomoda R., Nakajima T., Wake H. (1985). Photoelectrochemical Sterilization of Microbial Cells by Semiconductor Powders. FEMS Microbiol. Lett..

[91] Sunada K., Kikuchi Y., Hashimoto K., Fujishima A. (1998). Bactericidal and Detoxification Effects of TiO_2_ Thin Film Photocatalysts. Environ. Sci. Technol..

[92] Wei C., Lin W.-Y., Zainal Z., Zhu K., Smith R.L., Rajeshwar K. (1994). Bactericidal Activity of TiO_2_ Photocatalyst in Aqueous Media: Toward a Solar-Assisted Water Disinfection System. Environ. Sci. Technol..

[93] Wist J., Sanabria J., Dierolf C., Torres W., Pulgarin C. (2002). Evaluation of Photocatalytic Disinfection of Crude Water for Drinking-Water Production. J. Photochem. Photobiol. Chem..

[94] Cho G., Kwon J., Soh S.M., Jang H., Mitchell R.J. (2019). Sensitivity of predatory bacteria to different surfactants and their application to check bacterial predation. Appl. Microbiol. Biotechnol..

[95] Burmølle M., Webb J.S., Rao D., Hansen L.H., Sørensen S.J., Kjelleberg S. (2006). Enhanced Biofilm Formation and Increased Resistance to Antimicrobial Agents and Bacterial Invasion Are Caused by Synergistic Interactions in Multispecies Biofilms. Appl. Environ. Microbiol..

[96] Luppens S.B.I., Kara D., Bandounas L., Jonker M.J., Wittink F.R.A., Bruning O., Breit T.M., Ten Cate J.M., Crielaard W. (2008). Effect of Veillonella Parvula on the Antimicrobial Resistance and Gene Expression of *Streptococcus* Mutans Grown in a Dual-Species Biofilm. Oral Microbiol. Immunol..

[97] Simões M., Simões L.C., Vieira M.J. (2009). Species Association Increases Biofilm Resistance to Chemical and Mechanical Treatments. Water Res..

[98] Priyanka B., Patil R.K., Dwarakanath S. (2016). A Review on Detection Methods Used for Foodborne Pathogens. Indian J. Med. Res..

[99] Fu G., Vary P.S., Lin C.-T. (2005). Anatase TiO_2_ Nanocomposites for Antimicrobial Coatings. J. Phys. Chem. B.

[100] Achudhan D., Vijayakumar S., Malaikozhundan B., Divya M., Jothirajan M., Subbian K., González-Sánchez Z.I., Mahboob S., Al-Ghanim K.A., Vaseeharan B. (2020). The Antibacterial, Antibiofilm, Antifogging and Mosquitocidal Activities of Titanium Dioxide (TiO_2_) Nanoparticles Green-Synthesized Using Multiple Plants Extracts. J. Environ. Chem. Eng..

